# Quantification of Active and Latent Form of Human Cytomegalovirus Infection in Umbilical Cord Blood Donors by Real-Time PCR

**Published:** 2017-08-01

**Authors:** E. Abedi, M. Kheirandish, Z. Sharifi, S. Samiee, P. Kokhaei, Z. Pourpak, M. J. Ashraf

**Affiliations:** 1 Department of Immunology, Blood Transfusion Research Center, High Institute for Research and Education in Transfusion Medicine, Tehran, Iran; 2 Department of Microbiology, Blood Transfusion Research Center, High Institute for Research and Education in Transfusion Medicine , Tehran, Iran; 3 Department of Molecular Pathology, Blood Transfusion Research Center, High Institute for Research and Education in Transfusion Medicine. Tehran, Iran; 4 Department of Immunology, Semnan University of Medical Sciences, Semnan, Iran; 5 Department of Immunology, Asthma and Allergy Research Institute, Tehran University of Medical Sciences, Tehran, Iran; 6 Department of Pathology, Shiraz University of Medical Sciences, Shiraz, Iran

**Keywords:** Fetal blood, Cord blood stem cell Transplantation, Herpesviridae, Cytomegalovirus, Real-time polymerase chain reaction

## Abstract

**Background:**

Umbilical cord blood (UCB) is believed to be a highly valuable source of hematopoietic stem cells for transplantation.

Objective:

To investigate the prevalence of active and latent human cytomegalovirus (CMV) infection in UCB donors in Iranian population.

**Methods:**

A total of 825 UCB samples was collected under standard procedures and analyzed for the presence of CMV DNAs in buffy coat (latent infection) and plasma (active infection). DNA was extracted from buffy coat and plasma samples separately and tested with quantitative real-time PCR. All positive samples were checked by ELISA for IgG and IgM anti-CMV antibody.

**Results:**

Latent CMV infection was detected in 17 (2%) buffy coat samples with a low level of viral load, which indicated the presence of latent viral infection in donors. None of the plasma samples were found positive for CMV DNA reflecting no active infection. In the 17 positive samples, CMV viral load was 91–104 (mean: 100) copies/mL. All samples positive for viral DNA were also found positive for CMV IgG antibody by ELISA. No CMV IgM antibody was detected in positive samples.

**Conclusion:**

CMV is still the most important virus that infects hematopoietic stem cells and could be dangerous, especially for immunocompromized transplant recipients. We therefore suggest using real-time PCR for the detection and quantification of the viral DNA in buffy coat and plasma of UCB donors. PCR of plasma for detection of CMV and antibody assay for CMV infection add no more sensitivity for the detection of latent CMV infection in UCB donors.

## INTRODUCTION

The first-line treatment for various types of hematological malignancies, bone marrow failure and inherited immunodeficiency syndromes is transplantation of allogeneic hematopoietic stem cells (HSCs) [1, 2]. Umbilical cord blood (UCB) is believed to be a highly valuable source of hematopoietic stem cells for transplantation for lack of suitable, HLA-matched hematopoietic stem cell donors [1, 2]. Graft *vs*. host disease (GVHD) is considered one of the most important factors influencing transplant-related mortality. The less number of GVHD cases observed after UCB transplantation is probably a result of the naive condition of cord blood lymphocytes and the low cytotoxicity capabilities of cord blood T cells [1-3]. In any case of transplantation, infection is one of the major concerns. Among different infections in a immunocompromized patients, donor-transmitted infections are among the preventable causes of infections. UCB transplantation is associated with low risk of viral infection transmission, particularly with cytomegalovirus (CMV) and Epstein-Barr virus (EBV). The immaturity of immune cells in UCB can rise the risk of CMV infection after transplantation, particularly in adults [1]. The human CMV, human herpesvirus-5 (HHV-5) and two nearly related roseoloviruses, HHV-6 and HHV-7, are mentioned as the subfamily of the β-herpesvirinae. All of them are common pathogens and have a high seroprevalence in normal population [4-6]. The members of the β-herpesvirinae, similar to all other herpesviruses, can be reactivated from their persistent condition in immunocompromized hosts [7]. CMV persists without symptoms in an immunocompromized patient [6]. This virus can infect patients opportunistically after organ and HSC transplantation. CMV viral load is a risk factor for the development of CMV disease. 

CMV can infect almost all of population in developing countries and about 60% of people in developed nations [6]. CMV can be transmitted to a person by blood transmission. After usually a self-limited, subclinical primary infection, CMV persists in leuckocytes for lifelong in a latent form. This form has no clinical signs and symptoms, but it has the potential to be reactivated in an immunocompromized patient [8, 9]. Those with CMV IgG antibodies are at risk for reactivation of latent form of CMV. Reactivation of CMV may involve almost any organs, mainly kidney, lung, and the gastrointestinal tract [10]. In patients with hematological malignancies treated with conventional chemotherapy, CMV infection and reactivation are unusual infectious complications [10, 11]. The rate of CMV reactivation in stem cell transplant recipient is about 30%–70% [3]. 

Some investigators assume a potential rise in HHV-7 virulence with simultaneous CMV reactivation, that results in more severe CMV disease post-transplantation [12, 13]. Application of some methods similar to quantitative methods and biopsy for the detection of CMV has shown that CMV or other types of herpesvirus (HHV-6) are significantly associated with graft rejection after liver and kidney transplantation [14]. The objective of this study was to investigate the prevalence of active and latent CMV infection in UCB samples, an alternative source of HSC transplantation.

## MATERIALS AND METHODS

Clinical Specimens

Eight hundred and twenty-five pregnant women with a mean age of 26.5 years were included in this study. Informed written consent was taken from all participants, and UCB samples were obtained in Milad and Akabarabadi Hospitals, Tehran, and in Shoshtari Hospital, Shiraz, Iran.

We included all mothers aged between 17 and 35 years with a full-term pregnancy who accepted to participate in this study. The exclusion criteria were existence of any of following conditions: systemic diseases (e.g., diabetes mellitus, autoimmune conditions, etc.), infectious diseases (e.g., high risk patients for HIV infection or AIDS, malaria, fever in the past three years or taking anti-malaria drugs in the past six months, and presence of HCV antibody or HBs-Ag positivity), any history of malignancy, history of tattooing in the past year, and organ transplantation. 

DNA Extraction

A 10-mL blood sample was collected in a K2EDTA tube from 825 UCB samples. DNA was extracted from both buffy coat and plasma using high pure viral nucleic acid extraction kit (Roche, Germany) according to the manufacturer’s instructions.

Real-time PCR for CMV

Real-time PCR assay was carried out using SYBR Green Master mix reagents containing 5 µL extracted DNA added to 0.01% gelatin, 5 units Taq polymerase, 0.6 µM of forward primers (5’-GTGTGGGACATAGGCCAGAG-3’) and 0.6 µM of reverse primers (5’-GCGACATCCCCGCCTACTAC-3’) (201-bp product), 5 µL of reaction buffer (10 mM Tris-HCl, 50 mM KCl, pH 8.3), 200 µM of each deoxynucleotid triphosphate, following thermal profile optimized for real-time PCR as follows: 1 cycle of denaturation at 95 °C for 3 min, followed by 40 cycles of amplification at 95 °C for 15 sec, and 60 °C for 60 sec, using a thermal cycler (Rotor Gene 6000, Corbett Research). For each cycle during 60 °C extention, SYBR Green I fluorscence was detected and plotted using Rotor-gene 6000 series software (Corbett Research). The AcroMetrix^®^ CMVtc Panel (Invitrogen, Lifetechnologies, USA) was also used as a standard for preparing serial dilution in CMV-seronegative human plasma to concentration of 1×10^1^ to 1×10^6^ copies/mL. The limit of detection (LOD) in this assay using BBI (Boston Biomedical INC, USA) plasma was 50 copies/mL. A six-point standard curve as well as a positive and a negative control were also included in all runs.

Serological Assay

All plasma fractions of UCB samples with positive CMV DNA were screened for the detection of anti-CMV IgM and IgG antibody by valid semiquantitative ELISA kit (Trinity Biotech, USA).The experiment and cut-off calibration were conducted according to the manufacturer’s instructions.

## RESULTS

The study was performed on UCB samples of 825 full-term pregnant women with a mean age of 26.5 (range: 17–35) years. CMV DNA was detected in 17 (2%) buffy coat samples. None of the plasma samples were found positive for CMV DNA. In the 17 positive cases, CMV viral load ranged from 91 to 104 (mean: 100) copies/mL. All plasma fractions of UCB samples with positive CMV DNA were also found positive for Anti-CMV IgG Ab by ELISA. None of PCR-positive UCB samples were found positive for Anti-CMV IgM Ab.

## DISCUSSION

UCB transplantation is increasingly used in many centers because of the lower risk of GVHD compared with unrelated bone marrow transplantation. On the other hand, there is a concern of risk for opportunistic infection transmission by UCB.

Screening tests are performed for Treponema pallidum, hepatitis B (HBV) and C viruses, human T lymph tropic viruses 1 and 2, human immunodeficiency virus (HIV) 1 and 2, and recently acquired CMV. In transplant recipients, HHV is a commonly transmitted pathogen. To reduce the risk of CMV infection in cord blood transplantation, UCB donors are routinely screened for the presence of anti-CMV IgM and IgG. UCB donors are rejected if there is a serologic evidence of infection.

Infections with herpesviridae, especially with CMV, are the major problems in stem cell transplant recipients [15]. Weinberg, *et al*, in 2005 studied 362 randomly selected whole UCB aliquots and serum from UCB with multiplex PCR. The incidence of CMV DNA in these samples was zero, both in whole UCB and serum samples [16]. The results of this study showed that the incidence of CMV DNA in serum samples (active infection) was zero, though low level of CMV DNA in buffy coats of UCB samples was detected (2%). This reflects latent CMV infection in UCB donors.

Our results with ELISA were compatible with Monavari’s results in 2012 [17]. These showed poor sensitivity of IgM and IgG antibody assay for detection of active CMV infection. In the present study, we tested plasma and leukocyte for active and latent infections, separately. In addition to the detection method, type of blood components, plasma *vs*. peripheral blood leukocyte (PBL) may affect the positive rate of human CMV. The rate of PCR positivity for CMV in PBL is much higher because CMV harbors in PBL and we have a higher DNA concentration in PBL than that in serum [17, 18].

On the other hand, using PBL DNA in nucleic acid amplification assay for CMV is not suitable for the diagnosis of active CMV infection. Behbahani, *et al*, (2008) studied 30 allogenic bone marrow donors samples and 34 UCB HSCs by nested PCR. They found CMV DNA in 22 (73%) bone marrow progenitor cells and in 8 (24%) UCB samples [19]. The difference between our findings and theirs may be due to the larger sample size in our study and using standard donor selection criteria for UCB donors. In our study, HCMV was detected with real-time PCR, but Behbahani, *et al*, (2008) detected human CMV with nested PCR. Quantitative real-time PCR (qRT-PCR) is widely used for its advantages in quantifying the viral load and decreasing the probability of contamination in a closed system [20-24]. However, in nested PCR, false positive due to cross-contamination is a main problem [25]. Cross-contamination can occur at the time of sampling, extracting, and amplifying of DNA [26, 27]. The target population and other factors such as DNA extraction protocol and PCR primers may also influence the amplification efficiency [28].

Sassenscheidt, *et al*, in 2006 studied 198 plasma samples of 37 patients who underwent allogenic stem cell transplantation with 5-exonuclease (Taq man) quantitative real-time PCR. In their investigation, CMV was found in all samples that were seropositive for CMV. Their results suggest that CMV may be associated with post-transplantation end-organ disease [29]. 

Cahu, *et al*, (2009) studied 31 unrelated UCB transplant adult patients with hematological malignancies by PCR. They found recurrent CMV infection in 21% of the transplant recipients [30], which indicates the value of latent CMV detection in donor as a possible source of CMV reactivation

From the epidemiological point of view, determination of CMV positivity in this geographic area with large sample size can be a strong basic data for comparison of different studies (now and future) in the Middle Eastern countries and also other parts of the world. Determination of copy number of the virus, which deeply affects the post-transplantation events, is another novel point that should be considered in this investigation. 

This study conducted on a large number of UCB samples showed that the low rate of latent CMV infection in UCB samples could easily be overlooked with plasma CMV PCR analysis. IgG seropositivity seems to be not informative due to very high seropositivity in most populations. IgM antibody also has no role in detecting the latent infection (0% sensitivity in this study). However, the only reliable method to detect latent CMV infection in UCB samples is the real-time PCR on UCB buffy coat. Fortunately, CMV has a low prevalence. However, more studies are needed to determine the role of donor latent CMV infection *vs*. other possible sources of CMV infection in the pathogenesis of post-transplantation CMV-related complications. 

In conclusion, There have been significant strides in using UCB stem cells in cellular therapy because of their excellent therapeutic efficacy in bone marrow recovery and regenerative medicine. However, the necessity of increase of transplantation safety is recommended [31-35]. We believe that real-time PCR can detect and quantify CMV load and should be considered the standard analytical method for the molecular detection of CMV DNA in buffy coat and plasma of UCB donors. 
